# Subcutaneous administration of TC007 reduces disease severity in an animal model of SMA

**DOI:** 10.1186/1471-2202-10-142

**Published:** 2009-11-30

**Authors:** Virginia B Mattis, Marina Y Fosso, Cheng-Wei Chang, Christian L Lorson

**Affiliations:** 1 Department of Veterinary Pathobiology, Bond Life Sciences Center, University of Missouri, Columbia MO, USA; 2 Department of Chemistry and Biochemistry, Utah State University, Logan UT, USA

## Abstract

**Background:**

Spinal Muscular Atrophy (SMA) is the leading genetic cause of infantile death. It is caused by the loss of functional *Survival Motor Neuron 1 *(*SMN1*). There is a nearly identical copy gene, *SMN2*, but it is unable to rescue from disease due to an alternative splicing event that excises a necessary exon (exon 7) from the majority of *SMN2*-derived transcripts. While SMNΔ7 protein has severely reduced functionality, the exon 7 sequences may not be specifically required for all activities. Therefore, aminoglycoside antibiotics previously shown to suppress stop codon recognition and promote translation read-through have been examined to increase the length of the SMNΔ7 C-terminus.

**Results:**

Here we demonstrate that subcutaneous-administration of a read-through inducing compound (TC007) to an intermediate SMA model (*Smn*-/-; *SMN2*+/+; SMNΔ7) had beneficial effects on muscle fiber size and gross motor function.

**Conclusion:**

Delivery of the read-through inducing compound TC007 reduces the disease-associated phenotype in SMA mice, however, does not significantly extend survival.

## Background

Spinal Muscular Atrophy (SMA) is the leading genetic cause of infantile death. The clinical categorization of SMA is based upon disease severity and age of onset [1]. SMA results from the lack of a functional copy of the *Survival Motor Neuron 1 *(*SMN1*) gene [2]. There is a nearly identical copy gene, *SMN2*, found only in humans [3]. *SMN2 *does not fully compensate for the loss of *SMN1*, due to an alternative splicing event induced by a non-polymorphic C to T transition within exon 7 [4,5]. Alternative pre-mRNA splicing, therefore, results in the majority of *SMN2 *transcripts excluding exon 7 (SMNΔ7), encoding a less stable protein product compared to full-length SMN [6,7]. Loss of full-length SMN protein leads to the death of α-motor neurons, however, it is unclear why motor neurons are particularly susceptible to low levels of this ubiquitously expressed protein. However, two hypotheses suggest that axonal RNA transport and/or snRNP biogenesis underlie the SMA-associated activity [8].

Since the lack of SMN exon 7 leads to SMA development, this sequence is clearly important. There is a cytoplasmic localization signal within SMN exon 7 (QNQKE) that can transport SMN exons 1-6 and other heterologous nuclear proteins to the cytoplasm [9]. However, heterologous sequences can mediate proper SMN localization in several cellular contexts *in vitro*. In addition, heterologous sequences can also partially restore functionality in cell-based assays, such as protein stability, snRNP assembly and neurite extension assays [10-13]. For these reasons, aminoglycosides have been examined in SMA for their ability to induce translational "read-through." These compounds are presumed to inhibit the recognition of the endogenous SMNΔ7 translational stop codon, located four amino acids into exon 8. "Read-through" of the stop codon would thereby allow the incorporation of a C-terminal extension of an additional five amino acids [10-13]. Treating Type I primary SMA patient fibroblasts (3813s) or SMA iPS-derived neuronal cultures with aminoglycosides was shown to increase SMN protein [10,13,14]. When delivered via intra-peritoneal injection, treatment with the aminoglycoside G418 in a SMA mouse model increased gross motor function at select time points [10]. Additionally, direct injection of TC007, a novel aminoglycoside, into the central nervous system extends life span approximately 30% and elevates SMN protein in the brain and spinal cord [15]. To begin to examine a less invasive means of delivery, we have examined alternative routes of delivery for TC007. Here we demonstrate that subcutaneous administration of TC007, while not extending survival, results in improved molecular and cellular aspects of the SMA phenotype, as well as benefits in gross motor function in a SMA mouse model.

## Results and discussion

### Subcutaneous dosing of SMA mice with TC007 significantly increases gross motor function, but not lifespan

**Figure 1:**
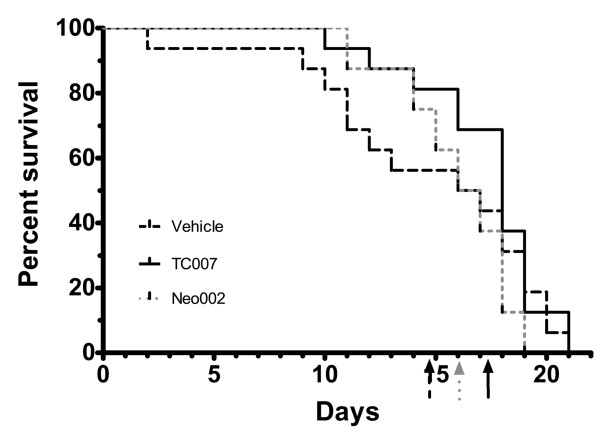
**TC007-treated mice have a slightly increased average lifespan**. Kaplan-Meyer shown of TC007-treated (solid black, n = 16), Vehicle-treated (PBS; dotted black, n = 16), and Neo002-treated (dotted-grey, n = 8) SMA mice lifespans. Arrows on X-axis represent the average lifespan. No significant difference was seen between the groups.

**Figure 2:**
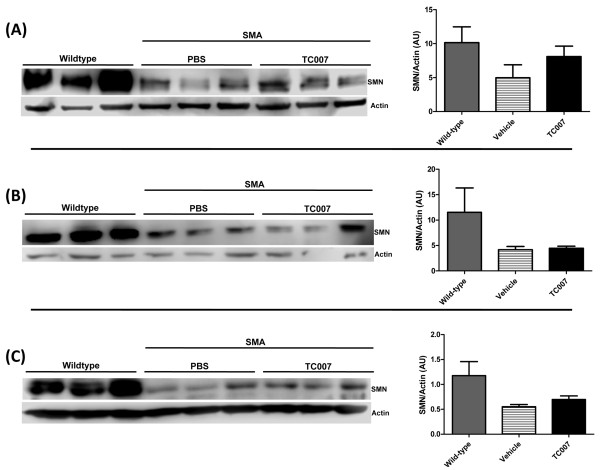
**No significant induction of SMN protein seen after subcutaneous TC007 administration**. Representative western blots and quantification shown of **(A) **Brain, **(B) **Spinal cord and **(C) **Triceps of TC007- (black bar) or vehicle-treated (striped bar) SMA mice and uninjected wild-type (grey bar) mice. Actin used as a loading control. Error bars represent SEM and an "n" of 3 was used for each group.

Based on previous *in vivo *examinations of aminoglycosides and stop codon readthrough [16], we administered subcutaneous doses of TC007 [17] at 30 mg/kg daily from post-natal day (p) 2 to 15 to the well-characterized Δ7 SMA model (*Smn*-/-; *SMN2*+/+; SMNΔ7) [18]. TC007 treated mice (n = 16) lived approximately 16% longer (from an average of 15.75 ± 1.315 to 18.25 ± 0.7555 days) than vehicle treated mice (n = 16), although this difference did not reach statistical significance (Figure 1). The aminoglycosides appeared to prevent the death at a younger age, resulting in larger numbers of mice dying later (median lifespan of vehicle-treated mice 17.5 days versus a median of 19 days for TC007-treated). However, the longest life spans were similar in the vehicle and TC007-treated mice. Moreover not all aminoglycosides that increase SMN levels confer the same survival effect. For example, administration of Neo002 (n = 8) [19], a compound containing the same chemical backbone as TC007, significantly elevated SMN levels *in vitro *(data not shown), but only marginally affected lifespan (Figure 1). Total body weight was unaffected in TC007 and Neo002 treated animals compared to vehicle treated SMA animals (data not shown). Since SMN read-through therapies are proposed to function in part through a stabilization of SMNΔ7 protein, various tissues were examined for an increase in steady-state levels of SMN protein. Therefore brain, spinal cord and muscle of p10-treated mice were isolated. Under these experimental conditions, we could not detect a significant increase of SMN (Figure 2), which may also explain the modest extension in lifespan.

**Figure 3:**
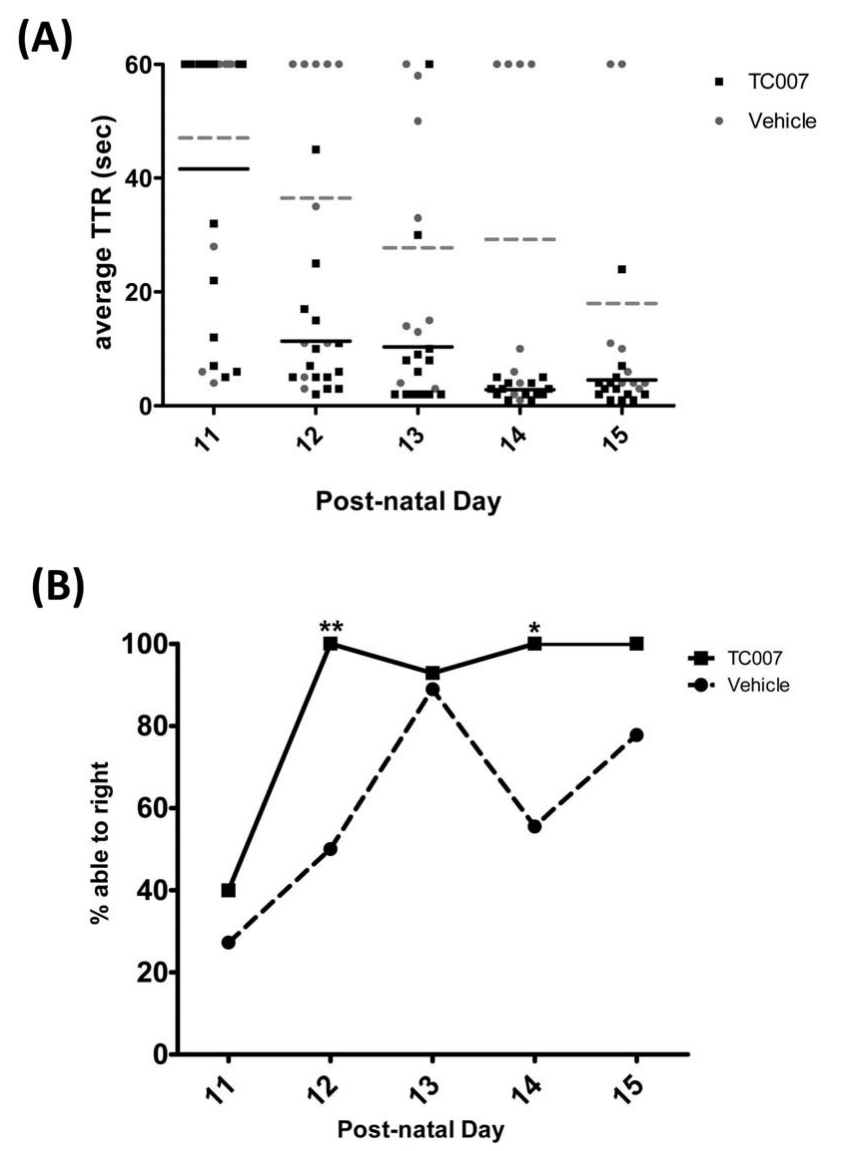
**TC007-treated mice have significantly increased gross motor function than vehicle treated as measured by time-to-right (TTR)**. **(A) **TC007-treated mice right faster than vehicle treated mice. Each circle (vehicle, n = 16) or square (TC007, n = 16) represents an individual mouse, with the bar (solid, TC007; dotted, vehicle) demonstrating the average. **(B) **More TC007-treated mice are able to right on average. Average percentage able to right plotted against day for each group. Black solid line represents TC007-treated group and grey dotted line represents vehicle-treated. Error bars represent SEM and significant days indicated by: *p < 0.05 or ** p < 0.02 as determined by two-way ANOVA.

**Figure 4:**
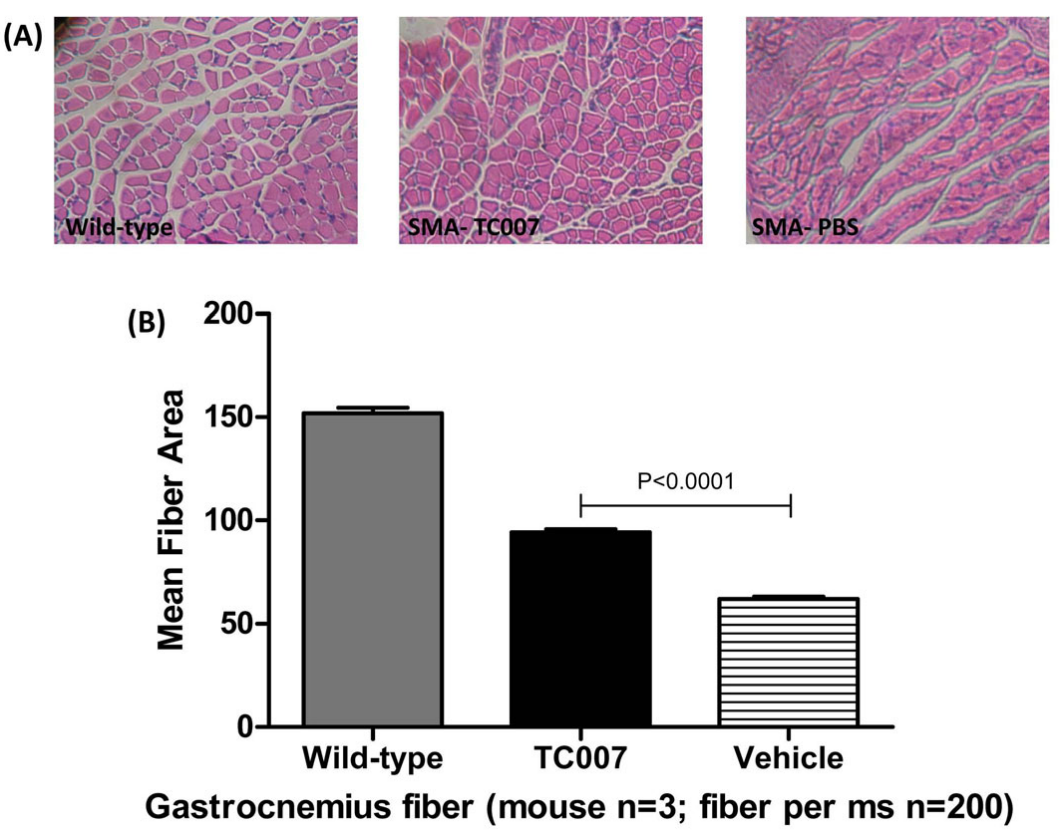
**Gastrocnemius muscle fibers from p10 mice were significantly larger in TC007-subcutaneously treated intermediate mice**. **(A) **Representative pictures of fixed and H&E stained gastrocnemius fibers from Wild-type or SMA mice (TC007- or vehicle-treated, respectively). **(B) **Graph of mean fiber area of the three groups. Significance by Student's T Test between TC007- or vehicle-treated mice indicated on graph (*), error bars represent SEM, and an "n" of 3 was used for each group.

TC007-treated mice, however, did exhibit increased gross motor function. A previously quantifiable measure of gross motor function in these SMA mice is determining the time it takes for a pup to right itself from a prone position, or time-to-right [20]. Not only did TC007-treated pups right faster, but they were also able to right significantly more often than their untreated counterparts at mid- to end-stage of disease (Figure 3A and 3B). When the gastrocnemius of p10 mice were examined, the fibers were significantly larger in the TC007-treated group (Figure 4), showing an anatomical correlation with our observed increase in overall gross motor function.

**Figure 5:**
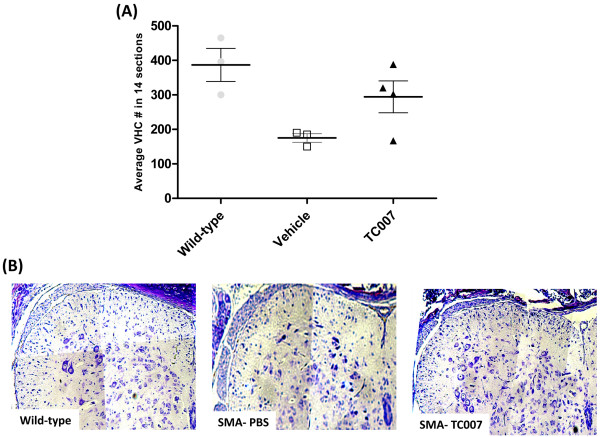
**Ventral horn cells (VHCs) trend towards increased VHC numbers between TC007- versus vehicle-treated SMA mice**. **(A) **Average VHC number in 14 sections of Wild-type, or vehicle- and TC007-treated p10 SMA mice. Each point represents an individual mouse, the horizontal line represents the average, and error bars represent SEM. **(B) **Representative pictures of fixed and cresyl violet-stained lumbar spinal cord sections from Wild-type or SMA mice (vehicle- or TC007-treated, respectively with an "n" of three for each group).

To determine if with the increase in fiber size, an increased survival of motor neurons was also observed, ventral horn cells (VHCs) were examined of p10 TC007-treated and vehicle-treated mice. While there was no increase in VHC cell body size, there was a slight trend towards an increased number of VHCs in treated tissues (Figure 5), although statistical significance was not achieved. This time point was chosen for histological examination due to its correlation with the most prominent display of an increase in gross motor function by the TC007-treated group. The increased fiber size along with the increase VHC numbers does provide a cellular corroboration to the improved gross motor phenotype seen upon subcutaneous administration of TC007. However, more pronounced improvements were likely not observed if TC007 failed to cross the blood-brain-barrier or if the compound exhibits a short half life. As no medicinal chemistry has been performed on TC007 to improve its drug-like qualities, it is not surprising that at this early stage the compound lacks properties typically associated with drugs for CNS disorders. To date, properties such as blood-brain-barrier permeability and pharmacokinetics have not been examined for TC007 but will be important to analyze in the event that TC007 or similar scaffolds move forward toward clinical application. While it is possible that TC007 is functioning as a general neuroprotectant, it is more likely that SMN levels were the basis for the cellular and gross motor activity increases. Unfortunately, the in vivo examination of this compound did not correlate with the in vitro activity when administered under these experimental conditions [14], however, direct administration of TC007 into the CNS by intraventricular injection did significantly elevate SMN levels and extend life by approximately 30% [15]. Clearly, moving this compound forward and making it more amenable for central nervous system diseases will require an in depth analysis of its pharmacokinetics and the development of medicinal chemistry beyond these proof-of-concept experiments.

### SMNΔ7 read-through benefits SMA mouse models

Translation stop codon read-through occurs at a relatively low frequency in the presence of aminoglycosides and this is likely true for the SMNΔ7 exon 8 stop codon based upon its specific stop codon and the proximal sequences flanking the stop signal [10,14]. Aminoglycoside treatment likely generates a low level of SMN read-through protein that enters into an SMN complexes with the existing full-length protein. This theory is supported by previously published data, demonstrating a small increase in SMN-FL protein after treatment, without increasing *SMN2*-transcription or altering exon 7 splicing [14]. *In vitro *studies have indicated that this possible hetero-oligomerization leads to an increase in the functionality of the SMN protein [12]. While it is still possible that read-through SMN is not as active in SMA-specific activities, experiments such as the development of a read-through SMA mouse would be an important step towards determining the extent of SMN read-through functionality and whether read-through based therapeutic strategies hold promise for SMA.

## Conclusion

In conclusion, similar benefits have been reported here for TC007 regarding a SMNΔ7 read-through therapy compared to previously published work using a FDA-approved aminoglycosides, G418 [10]. While TC007 treatment does not result in a statistically significant extension in median lifespan, there were significant increases in gross motor function and muscle integrity at mid-stage of disease (p10). Extension in life span was demonstrated when TC007 was administered directly to the central nervous system via intracerebral ventricular injection, suggesting that the importance in delivery strategies as well as likely target tissues for SMA therapeutics [15]. Taken together, these results demonstrate the proof-of-concept utility of read-through compounds in SMA cell and animal models.

## Methods

### Animals and drug treatment

All animal experiments were carried out in accordance with protocols approved by the Animal Care and Use Committee of the University of Missouri. Mice were genotyped and litters excluded as previously described [21]. TC007 was initially resuspended in dH_2_0, further diluted in PBS, and administered by subcutaneous injection (10 μl/gram of body weight) on post-natal days 2 through 15. PBS (vehicle) was injected as a negative control. To assess gross motor function, righting reflex (as described in [20]) was measured starting at post-natal day 5.

### Western blot analysis

Tissues were harvested at indicated times and analysis was performed as previously described [21]. Mouse anti-SMN (BD) was used for western at 1:1,000 and rabbit anti-actin (Sigma) was used at 1:250.

### Histology and morphometry

Spinal cords were harvested on post-natal day (p) 10 and analysis of VHCs were performed as previously described [21]. Gastrocnemius muscle was also harvested on p10, dissected, fixed in 4% paraformaldehyde, embedded in paraffin, sectioned and stained with hematoxylin and eosin stain. 200 fiber diameters were measured per section, 15 sections per muscle.

### Statistical analyses

Analysis was performed as previously described [21]. Briefly, error bars on graphs represent Standard Error of the Mean (S.E.M.). Significance of lifespan between TC007- and vehicle-treated mice was determined by Mantel-Cox test. All other indicated statistical significance was calculated by either Student's T Test or two-way ANOVA with a Bonferonni *post hoc *test of p < 0.05 or greater, as indicated in figure legend.

## Abbreviations

(SMN): Survival Motor Neuron; (SMA): Spinal Muscular Atrophy; (p): post-natal day; (VHC): ventral horn cell.

## Authors' contributions

VBM carried out all mouse and molecular studies, participated in the design of the study and drafted the manuscript. C-WC and MF manufactured compounds TC007 and Neo002. CLL conceived of the study, and participated in its design and coordination and drafted the manuscript. All authors read and approved the final manuscript.
